# Identification of N6-Methyladenosine-Related lncRNAs as a Prognostic Signature in Glioma

**DOI:** 10.3389/fonc.2022.789283

**Published:** 2022-03-03

**Authors:** Yujia Chen, Yuduo Guo, Shenglun Li, Jiacheng Xu, Xiang Wang, Weihai Ning, Lixin Ma, Yanming Qu, Mingshan Zhang, Hongwei Zhang

**Affiliations:** ^1^ Department of Neurosurgery, Sanbo Brain Hospital, Capital Medical University, Beijing, China; ^2^ CAS Key Laboratory of Infection and Immunity, Institute of Biophysics, Chinese Academy of Sciences, Beijing, China

**Keywords:** The Cancer Genome Atlas, N6-methyladenosine, long non-coding RNA, prognostic signature, glioma

## Abstract

N6-methyladenosine (m6A) modification is the most abundant modification in long noncoding RNAs (lncRNAs). Current studies have shown that the abnormal expression of m6A-related genes is closely associated with the tumorigenesis and progression of glioma. However, the role of m6A-related lncRNAs in glioma development is still unclear. Herein, we screened 566 m6A-related lncRNAs in glioma from The Cancer Genome Atlas (TCGA) database. The expression pattern of these lncRNAs could cluster samples into two groups, in which various classical tumor-related functions and the tumor immune microenvironment were significantly different. Subsequently, a nine-factor m6A-related lncRNA prognostic signature (MLPS) was constructed by using a LASSO regression analysis in the training set and was validated in the test set and independent datasets. The AUC values of the MLPS were 0.881, 0.918 and 0.887 for 1-, 3- and 5-year survival in the training set, respectively, and 0.856, 0.916 and 0.909 for 1-, 3-, and 5-year survival in the test set, respectively. Stratification analyses of the MLPS illustrated its prognostic performance in gliomas with different characteristics. Correlation analyses showed that the infiltrations of monocytes and tumor-associated macrophages (TAMs) were significantly relevant to the risk score in the MLPS. Moreover, we detected the expression of four MLPS factors with defined sequences in glioma and normal cells by using RT–PCR. Afterwards, we investigated the functions of LNCTAM34A (one of the MLPS factors) in glioma cells, which have rarely been reported. *Via in vitro experiments*, LNCTAM34A was demonstrated to promote the proliferation, migration and epithelial-mesenchymal transition (EMT) of glioma cells. Overall, our study revealed the critical role of m6A-related lncRNAs in glioma and elucidated that LNCTAM34A could promote glioma proliferation, migration and EMT.

## Introduction

Glioma is the most common malignant primary intracranial tumor, and it possesses a high recurrence rate and mortality (1). Due to its high heterogeneity, the currently recommended maximum surgical resection (combined with radiotherapy and chemotherapy) cannot completely cure this tumor. In addition, the malignancy of gliomas is progressive, and more than half of lower grade gliomas (LGG) can evolve to higher grade gliomas and develop resistance to chemotherapy. Therefore, it is urgent to identify new therapeutic targets and prognostic indicators for glioma. In recent years, an increasing number of biomarkers, such as the isocitrate dehydrogenase (IDH) mutation and the codeletion of chromosome arms 1p and 19q (1p/19q codeletion), have been integrated into the 2016 WHO classification to illustrate histological features (2).

Long noncoding RNA (lncRNA) is a type of noncoding RNA with a length greater than 200 nt that can regulate gene expression at the transcriptional and posttranscriptional levels *via* multiple mechanisms (3). Previous studies have shown that the dysregulation of specific lncRNAs is closely related to the occurrence and development of various tumors. For example, lncRNA PVT1 was reported to facilitate the tumorigenesis and progression of glioma (4). Moreover, lncRNA ATB has been shown to promote TGF-β-induced glioma cell invasion through the NF-κB and P38/MAPK pathways (5). Additionally, lncRNA BCYRN1 has been found to inhibit glioma tumorigenesis by competitively binding with miR-619-5p to regulate CUEDC2 expression and the PTEN/AKT/p21 pathway (6).

N6-methyladenosine (m6A) is a chemical modification present in multiple RNA species, including lncRNAs (7). It was discovered in the 1970s, and studies have increased in recent years (8–11). The modification of m6A is regulated by a series of factors, including methyltransferases, binding proteins and demethylases, which are also known as writers, readers and erasers (12, 13). Many reports have identified their essential roles in physiological processes, and some studies have shown that dysregulation of m6A regulatory factors may be related to the malignant development of glioma (14–17).

Currently, we have a further understanding of m6A and lncRNAs in tumors, but the role of m6A-related lncRNAs in glioma is still unclear. Few studies have analysed the potential role and mechanisms of m6A-related lncRNAs in specific glioma subtypes, thus limiting the generalizability of these results in gliomas (18, 19). Thus, a comprehensive understanding of the role of m6A-related lncRNAs in glioma remains to be developed.

In this study, we elucidated the critical role of m6A-related lncRNAs by analyzing their expression profile in glioma and patient prognoses, and we developed a m6A-related lncRNA prognostic signature (MLPS). Moreover, from this signature, we detected the expression of four lncRNAs with defined sequences in three glioma and one normal astrocyte cell lines. We also demonstrated the role of LNCTAM34A in promoting glioma proliferation, migration and epithelial-mesenchymal transition (EMT).

To date, this is the first m6A-related lncRNA prognostic signature to be used for all glioma patients, regardless of the differences in sex, age, World Health Organization (WHO) grade, IDH status or 1p19q deletion status. Moreover, this is the first study to clarify the glioma-promoting role of LCTAM34A. Our study expands the understanding of m6A-related lncRNAs in glioma and provides insight for further research.

## Results

### Screening and Clustering of m6A-Related lncRNAs in Gliomas

**Figure 1:**
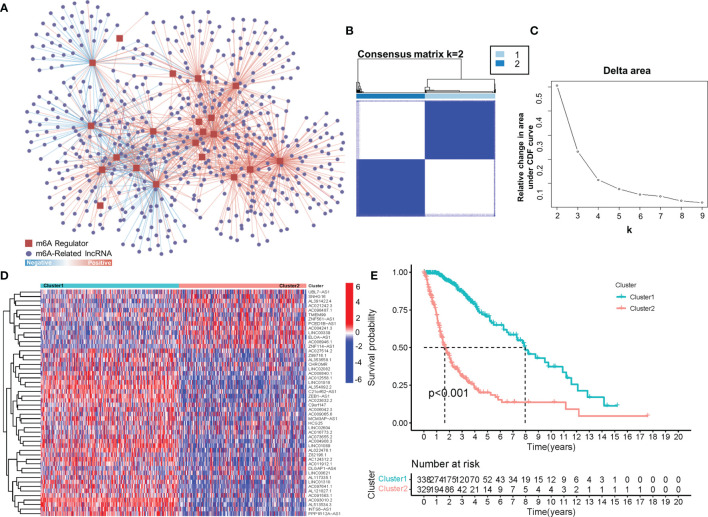
**(A)** The coexpression network of m6A regulators and m6A-related lncRNAs. **(B)** Consensus matrix of the consensus clustering based on the m6A-related lncRNAs for optimal k = 2. **(C)** Area under the CDF curve increment for k = 2 to 9. **(D)** The heatmap of 50 randomly m6A-related lncRNAs in 2 clusters. **(E)** Overall survival of patients (TCGA) in different clusters.

To obtain lncRNAs related to m6A regulation, we collected 23 m6A regulators from previous studies, including eight m6A writers, 13 readers and two erasers (**Table S1**) (18, 20). Afterwards, by using a correlation analysis, 566 m6A-related lncRNAs (|cor value|>0.4, p<0.001) were screened from 14,086 lncRNAs. As shown in **Figure 1A**, hundreds of lncRNAs were suggested to be involved in m6A regulation, thus implying a potential role for m6A-related lncRNAs in glioma. Furthermore, consensus clustering was performed to clarify the expression characteristics of m6A-related lncRNAs in gliomas. The cumulative distribution function (CDF) of the consensus cluster from k=2 to 9 and the increment in the AUC were analysed. When the consensus matrix k was 2, there existed the least crossover between the glioma samples and the maximum increment of the area under the CDF curve; thus, k=2 was determined (**Figures 1B, C**). To visualize the expression pattern of m6A-related lncRNAs between the two clusters, we plotted a heatmap of 50 randomly selected m6A-related lncRNAs (**Figure 1D**), which exhibited a significant difference in the m6A-related lncRNAs between the two clusters. Moreover, as shown in **Figure 1E**, the prognosis in Cluster 2 was significantly better than that in Cluster 1. These results suggested the heterogeneous and prognostic values of m6A-related lncRNAs in gliomas.

### Immune Landscape of Gliomas in Different Clusters

**Figure 2:**
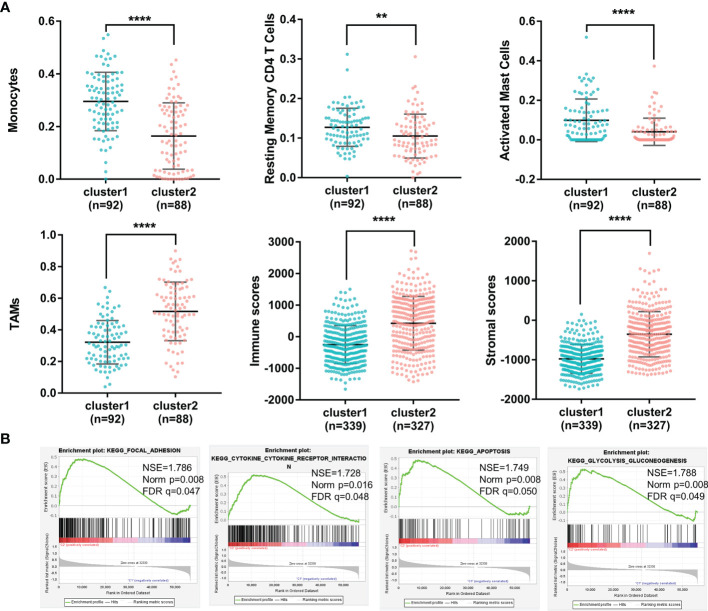
**(A)** The percentages of monocytes, resting memory CD4 T cells, activated mast cells, TAMs, immune scores and stromal scores of gliomas in the two clusters (****p < 0.0001, **p < 0.01). **(B)** Different functional pathway enrichments in the two clusters.

Due to the fact that the importance of immune regulation in tumors has been proven (21–25), we profiled the immune landscape of m6A-related lncRNAs in gliomas by using the CIBERSORT and ESTIMATE algorithms. Fifteen of the 22 tumor-infiltrating immune cells differed in these two clusters (**Figure S1**). Among them, immune cells with a proportion higher than 5% showed significant differences in the groups. As shown in **Figure 2A**, levels of monocytes, resting memory CD4 T cells and activated mast cells were higher in Cluster 1 (with a better OS) than in Cluster 2 (with a worse OS). In contrast, TAMs were significantly higher in Cluster 2 than in Cluster 1. In addition, the immune scores and stromal scores were significantly different between Cluster 1 and Cluster 2, as calculated by the ESTIMATE algorithm (**Figure 2A**). Cluster 2 had higher immune scores and higher stromal scores than Cluster 1. These results demonstrated glioma immune heterogeneity and suggested a correlation between m6A-related lncRNAs and the tumor immune microenvironment.

### GSEA of m6A-Related lncRNAs in Gliomas

To investigate the potential biological processes involved in m6A-related lncRNAs, a gene set enrichment analysis (GSEA) was performed between Cluster 1 and Cluster 2. It revealed that 21 gene sets were significantly enriched in Cluster 2 compared with Cluster 1 (p<0.01) (**Supplementary 2**). Among them, four hallmarks were significantly enriched in Cluster 2 (FDR<0.05), including the focal adhesion pathway, cytokine and cytokine receptor interaction pathway, apoptosis pathway and glycolysis pathway (**Figure 2B**). This result indicated that m6A-related lncRNAs may affect glioma progression by regulating specific pathways and further illustrated the significance of m6A-related lncRNAs in gliomas.

### Construction of the m6A-Related lncRNA Prognostic Signature (MLPS) From the Training Set

**Figure 3:**
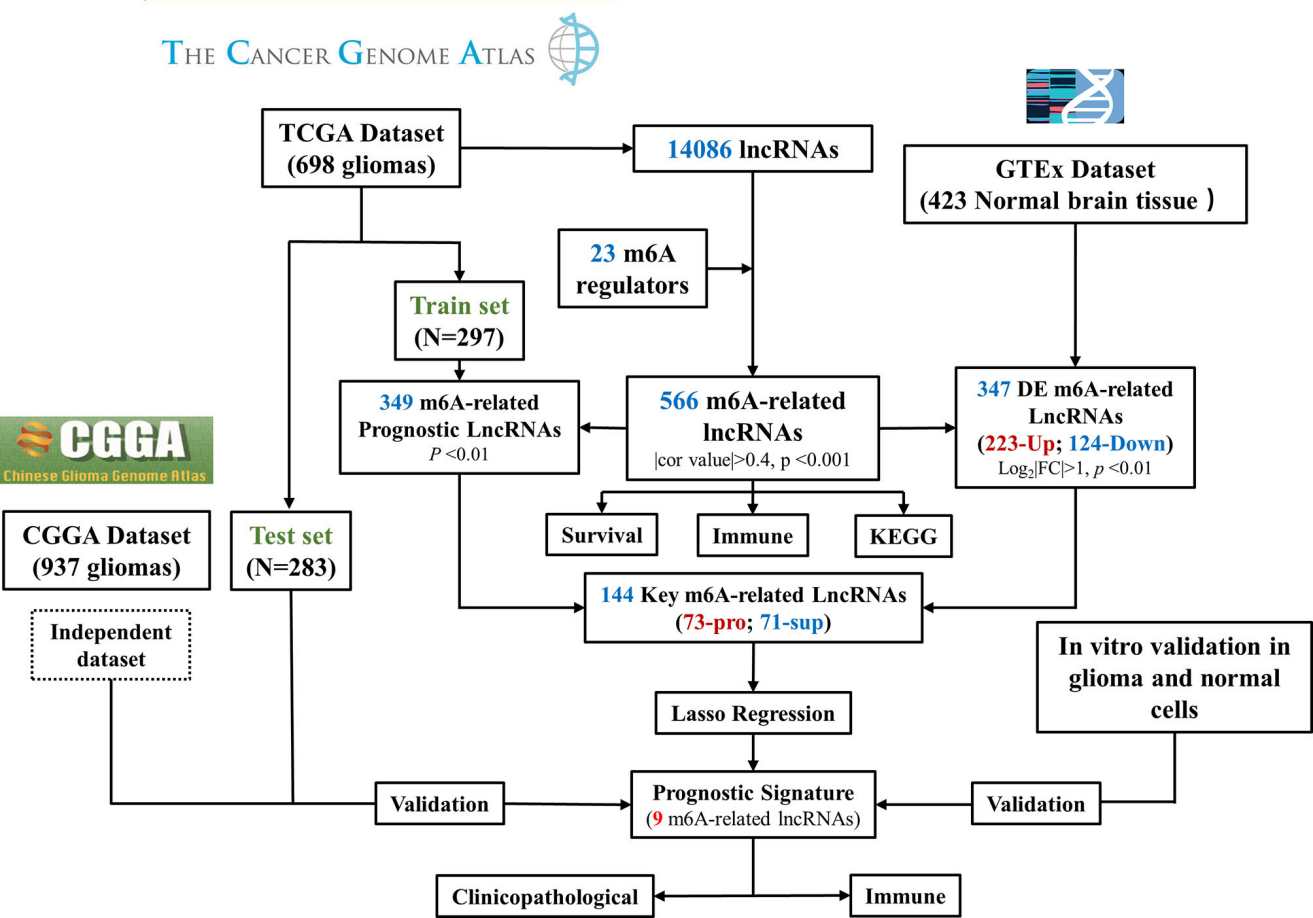
Flow chart of this study. DE, differentially expressed; Pro, promotor; Sup, suppressor. RT–PCR Quantitative Real-Time Polymerase Chain Reaction.

**Figure 4:**
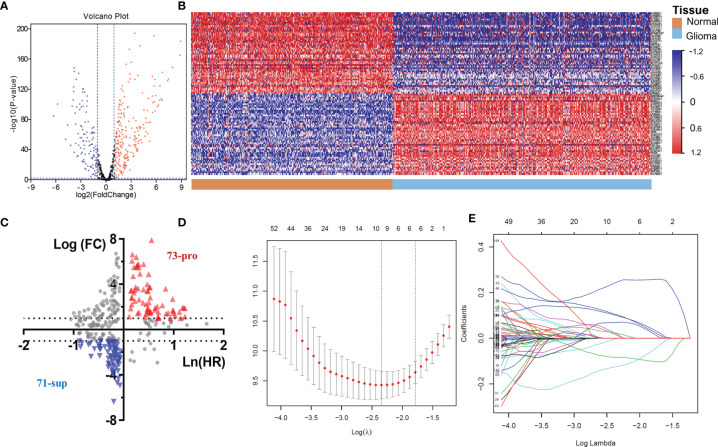
**(A)** The volcano plot of m6A-related lncRNAs in glioma. **(B)** Heatmap of the 100 top differentially expressed m6A-related lncRNAs. **(C)** The 144 key m6A-related lncRNAs. **(D-E)** LASSO Cox regression analysis of m6A-related lncRNAs.

The above results demonstrated the vital role of m6A-related lncRNAs in glioma. Herein, we tried to develop a m6A-related lncRNA prognostic signature (MLPS) to identify key factors from the large number of m6A-related lncRNAs and to assess glioma patient prognosis accordingly. As shown in **Table 1**, the TCGA dataset was randomly divided into the following two subsets: the training set (n=297) and the test set (n=283), with no significant difference in age, sex, WHO grade, IDH status or 1p/19q status. Next, 347 DE m6A-related lncRNAs (|log2FC|>1 and p value<0.01) were obtained by comparing gliomas in the training set to normal brain tissues from the GTEx dataset (**Figures 3** and **4A, B**). Furthermore, we performed a correlation analysis between the prognosis of patients and the expression of m6A-related lncRNAs in the training set and found 349 m6A-related prognostic lncRNAs. Subsequently, 73 m6A-related lncRNAs with ln(HR)>0 and log2FC>1 were determined to be promoters, whereas 71 m6A-related lncRNAs with ln(HR)<0 and log2FC<-1 were considered to be suppressors (**Figure 4C**). Afterwards, *via* the LASSO regression analysis, nine m6A-related lncRNAs were identified to construct the prognostic signature from 144 key m6A-related lncRNAs, including AL390755.1, AL445524.1, AL359643.3, LINC00641, AL117332.1, LNCTAM34A, CRNDE, AP001486.2 and CARD8. AS1 (**Figures 4D, E**). The coefficients of these lncRNAs were used to calculate the risk score.


$$\begin{array}{ccc} Risk\;score=\text{AL}390755.1\times (0.0986)+\text{AL}445524.1\times (0.2462)+\text{AL}359643.3\times (0.0173) & +\text{LINC}00641\times (-0.0855)+\text{AL}117332.1\times (0.0125)+\text{LNCTAM}34\text{A}\times (0.0217) & +\text{CRNDE}\times (0.1025)+\text{AP}001486.2\times (-0.1237)+\text{CARD}8.\text{AS}1\times (0.0699) \end{array}$$

**Table 1:** The clinicopathological features of glioma patients in the training set and test set.

Datasets		Train set	Test set	p value
		(n = 297)	(n = 283)	
**Age** (mean ± SD, years)		46.61 ± 14.85	47.91 ± 16.03	0.311
**Survival time** (mean ± SD, years)		1.80 ± 2.25	1.74 ± 2.28	0.754
**Censor** (n)	Alive	91	81	0.595
	Dead	206	202
**Gender** (n)	Male	127	115	0.604
	Female	170	168
**WHO Grade** (n)	Lower grade (WHO I-II)	108	100	0.796
	Higher grade (WHO III-IV)	189	183
**IDH State** (n)	Mutant	187	177	0.917
	Wildtype	110	106
**1p/19q State** (n)	Codeletion	76	72	0.968
	Noncodeletion	221	211

A positive coefficient indicated that it was a risk factor, whereas a negative coefficient indicated a protective factor in glioma.

**Figure 5:**
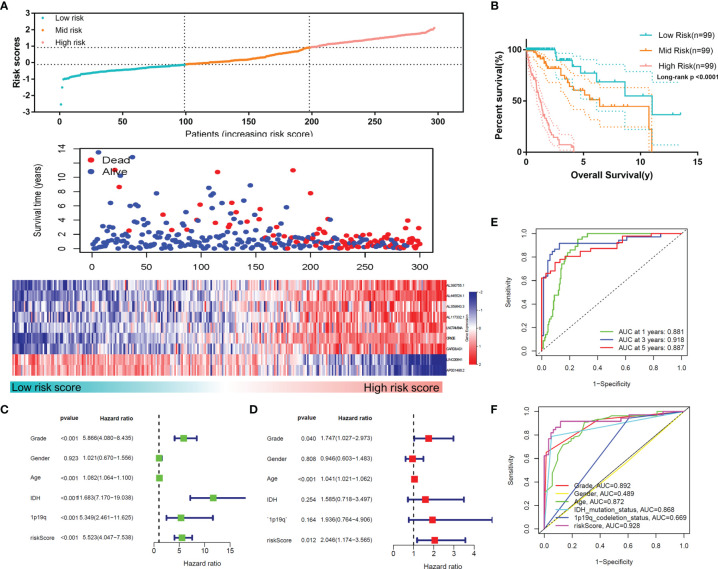
**(A)** The distributions of risk scores, alive/dead status and the expression of nine m6A-related lncRNAs in the training set. **(B)** Overall survival analysis for patients in the high- and mid-/low-risk groups. **(C)** Univariate and **(D)** multivariate Cox regression analyses for OS in glioma patients in the training set. **(E)** ROC curve of the risk score at different follow-up times. **(F)** The ROC curve of the risk score and other clinical characteristics.

According to this risk score, we divided the samples of the training set into low-, mid- and high-risk groups, and the expression profiles of the lncRNAs constituting the signature were found to differ markedly (**Figure 5A**). **Figures 5A, B** shows that among these three groups, a higher risk score indicated a worsened prognosis of the patients (p<0.0001), including the survival rate and survival time. The univariate and multivariate Cox regression analyses demonstrated the independent prognostic value of the MLPS in the training set (**Figures 5C, D**). The ROC curves showed that the MLPS had a robust predictive ability, with AUCs of 0.881, 0.918 and 0.887 for 1-, 3- and 5-year survival, respectively (**Figure 5E**). Its predictive performance was better (AUC=0.928) than other classic indicators, including age, sex, WHO grade, IDH mutation status and 1p/19q codeletion status (**Figure 5F**). These results indicated that the prognostic signature generated by m6A-related lncRNAs may serve as an efficient and accurate indicator for evaluating prognoses and suggested the potential role of these lncRNAs in gliomas.

### Validation of MLPS in the Test Set and CGGA Datasets

**Figure 6:**
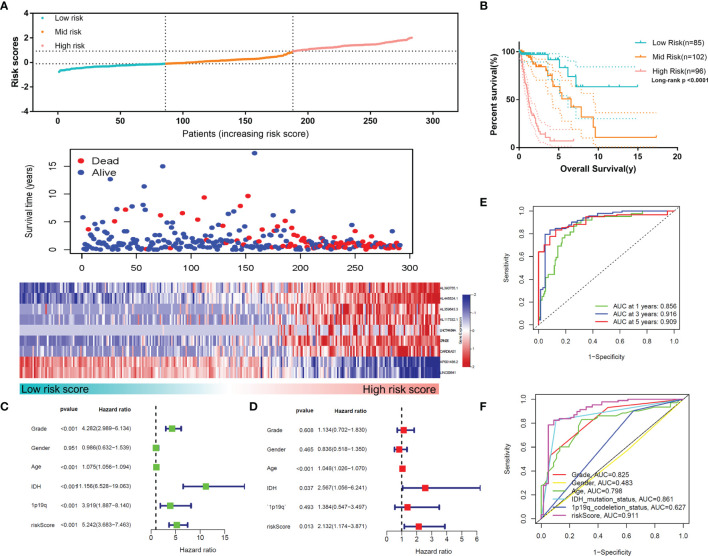
**(A)** The distributions of risk scores, alive/dead status and the expression of nine m6A-related lncRNAs in the test set. **(B)** Overall survival analysis for patients in the high- and mid-/low-risk groups. **(C)** Univariate Cox regression analyses and **(D)** multivariate Cox regression analyses for OS in glioma patients in the test set. **(E)** ROC curve of the risk score at different follow-up times. **(F)** The ROC curve of the risk score and other clinical characteristics.

To confirm the reliability of the MLPS, we used the test set for validation. As shown in **Figures 6A, B**, the high-risk group had the worst survival rate and time, and the low-risk group had the best survival rate and time in the test set (p<0.0001), which was consistent with the results in the training set. The univariate and multivariate Cox regression analyses demonstrated the independent prognostic value of the MLPS in the test set (**Figures 6C, D**). The ROC curves showed that the AUCs were 0.856, 0.916 and 0.909 for 1-, 3- and 5-year survival, respectively, and the AUC of the risk score was higher than that of the other indicators (**Figures 6E, F**). These results showed that this MLPS did have an excellent predictive effect on the prognosis of glioma patients.

To further verify the reliability of MLPS, we collected other independent datasets (CGGA-seq-1 and CGGA-seq-2) for further validation. Two lncRNAs of the signature (CRNDE and LINC00641) were found in these independent datasets. The survival curve showed that the survival of patients with high CRNDE expression was significantly worse than that of patients with low CRNDE expression, which was observed both in primary glioma patients and recurrent glioma patients (**Figure S3A, B**). In contrast, LINC00641 exhibited the opposite results of CRNDE in glioma. The survival curve showed that patients with high LINC00641 expression had significantly better survival than patients with low CRNDE expression (**Figure S3C, D**). In addition, the expression of CRNDE was significantly higher in glioma patients with high grade, IDH wildtype, 1p/19q noncodeletion or older age. In comparison, the expression of LINC00641 was higher in glioma patients with lower grades, IDH mutation, 1p/19q codeletion and younger age (**Figure S2**). These results suggested the clinical value and potential role of lncRNAs composing the signature in gliomas.

### MLPS as an Independent Prognostic Factor for Glioma Patients

**Figure 7:**
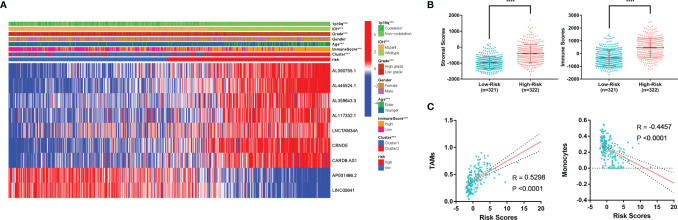
**(A)** Heatmap of MLPS and clinicopathological features. **(B)** Differences in stromal scores and immune scores in the high- and low-risk groups. **(C)** The correlation of immune cells and risk score. MLPS, m6A-related lncRNA prognostic signature; ****p < 0.0001.

To explore the relationship between the m6A-related lncRNA signature and clinicopathological features of glioma, we analysed the risk score in different glioma subgroups (**Figure 7A**). The results showed that there was no significant difference in the score among White, Asian or Black and African patients (**Figure S3E**). In addition, the results showed that high-risk glioma patients had a significantly worse OS in both the younger subgroup (age ≤ 60) and the older subgroup (age>60). Likewise, high-risk patients had a significantly worse OS than low-risk patients in the male or female subgroups, IDH mutant or wild-type subgroups, lower-grade (WHO I-II) or higher-grade (WHO III-IV) subgroups and 1p/19q codeletion or noncodeletion subgroups (**Figure S3F**). These results indicated that this MLPS was effective in predicting the prognosis of glioma patients with different clinicopathological features.

In addition, we found that the high-risk group had significantly higher stromal scores and immune scores than the low-risk group (**Figure 7B**), which implied a potential correlation between the MLPS and the tumor microenvironment of glioma. We screened the immune cell types with more than a 5 percent proportion in all immune cells and analysed their correlations with the risk scores. Two types of immune cells had significant correlations with the risk scores (|R|>0.4) (**Figure 7C**). The correlation analyses showed that monocytes were negatively correlated with the risk score, whereas TAMs were positively correlated with the risk score. These results suggested a potential correlation between the m6A-related lncRNA signature and the distribution of specific immune cells. In recognition of the favourable predictive effect of this signature, there may be a regulation underlying m6A-related lncRNAs and immune cells, which could jointly affect the malignancy of glioma.

### Validation of MLPS Factors *In Vitro*

**Figure 8:**
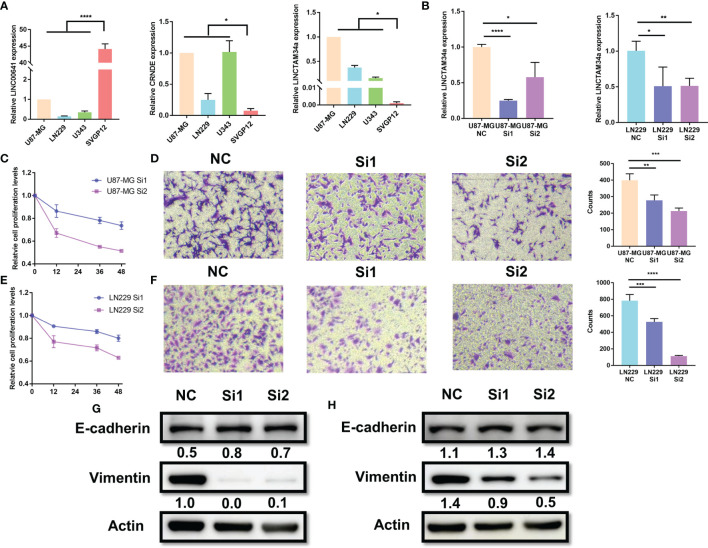
Knockdown of LNCTAM34A reduced proliferation, migration and EMT in glioma cells. **(A)** The expression levels of CRNDE, LINC00641 and LNCTAM34a in glioma cell lines (U87-MG, LN229 and U343) and normal human astrocytes (SVGP12) as detected by RT–PCR. **(B)** LNCTAM34A expression levels in U87-MG cells or LN229 cells transfected with siNC or two different specific siRNAs. The CCK-8 assay was used to detect **(C)** U87-MG cell or **(E)** LN229 cell proliferation at 0, 12, 36 and 48 h after incubation.The Transwell assay was used to detect **(D)** U87-MG cells or **(F)** LN229 cell migration at 24 h after incubation. **(G)** The expression of E-cadherin and Vimentin in U87-MG cells and **(H)** LN229 cells was measured by using Western blotting. ****p < 0.0001, ***p < 0.001, **p < 0.01, *p < 0.05.

Among the nine factors of the MLPS, four lncRNAs (CRNDE, LINC00641, LNCTAM34a and CARD8-AS1) with defined sequences were selected for further *in vitro* experiments. We detected their expression levels in glioma cell lines (U87, LN229 and U343) and a normal human astrocyte line (SVGp12) by using RT–PCR. We found that the expression of LINC00641 in glioma cells was significantly lower than that in normal astrocytes, and the expression of CRNDE and LNCTAM34a was significantly higher in glioma cells (**Figure 8A**). These results supported the above analysis and verified the validity of the prognostic signature. However, the expression of CARD8-AS1 was inconsistent with the results from the analysis. Specifically, it was expressed at higher levels in normal cells than in glioma cells, which may be attributed to the small number of cell lines included in this study (results not shown).

Among the four markers mentioned above, previous studies have identified that CRNDE and CARD8-AS1 are risk factors, and LINC00641 is a protective factor for glioma, which is consistent with our study results. However, the role of LNCTAM34A in glioma is still unclear. Therefore, we applied a series of experiments to identify the function of LNCTAM34A in glioma. We knocked down LNCTAM34A by using specific siRNAs in U87-MG and LN229 cells and detected LNCTAM34A expression by using RT–PCR (**Figure 8B**). Subsequently, we tested cell proliferation, migration and epithelial-mesenchymal transition (EMT) in normal control glioma cells and LNCTAM34A knockdown glioma cells. The CCK8 assay showed that the proliferation of U87 and LN229 glioma cells was significantly reduced after LNCTAM34A knockdown (**Figures 8C, E**), and the Transwell assay showed that the migration of glioma cells was also significantly reduced after LNCTAM34A knockdown (**Figures 8D, F**), thus demonstrating that LNCTAM34A could promote glioma tumor proliferation and migration. A Western blot analysis showed that E-cadherin was upregulated, but Vimentin was downregulated, in LNCTAM34A knockdown cells, thus proving that LNCTAM34A could regulate the EMT process in glioma cells (**Figures 8G, H**). All of the results showed that LNCTAM34A was a risk factor for glioma, which was consistent with MLPS.

## Discussion

Glioma is the most common primary malignant tumor of the central nervous system. The prognosis of patients is usually poor, and there is a lack of effective prognostic signatures. M6A is one of the most common modifications in lncRNAs. Some studies have shown that abnormal expression of m6A regulators is related to tumor occurrence and progression, but the manner in which it acts in a lncRNA-dependent manner during glioma progression is still unclear.

Herein, a total of 698 glioma patients from the TCGA database were included in this study to explore the prognostic signature of m6A-related lncRNAs. A nine-factor MLPS was constructed by selecting m6A-related prognostic lncRNAs and performing least absolute shrinkage and selection operator (LASSO) Cox regression analyses. The signature showed excellent predictive ability in multiple datasets and different stratifications of glioma patients. To validate the signature, we examined the expression levels of four lncRNAs in glioma and normal astrocytes. Moreover, *in vitro* experiments demonstrated the functions of LNCTAM34A in glioma, which could promote glioma proliferation, migration and EMT. At present, this is the first study to demonstrate the role of LNCTAM34A in glioma.

We identified 144 key m6A-related lncRNAs, nine of which were included in the MLPS. LINC00641 was reported to be an oncogene in acute myeloid leukaemia (26), colorectal carcinoma (27) and gastric carcinoma (28). Conversely, LINC00641 has been studied and reported as a tumor inhibitor in many cancers, including prostate cancer (29), bladder cancer (30), renal cancer (31), cervical cancer (32), non-small-cell lung cancer (33) and breast cancer (34). Additionally, LINC00641 has been shown to inhibit breast cancer cell proliferation, migration and invasion by sponging miR-194-5p (34). It prevented cell proliferation and enhanced cell apoptosis by targeting the miR-4262/NRGN axis in glioma (35). In our study, the expression of LINC00641 had a positive correlation with the OS of glioma patients, and the expression of LINC00641 was significantly higher in normal astrocytes than in glioma cells, which supported the idea that LINC00641 was a protective factor for glioma. CARD8-AS1 was identified as a risk lncRNA in glioma and ovarian cancer *via* a bioinformatic analysis (36, 37). CARD8-AS1 has been shown to promote the metastasis of glioma cells *in vitro (* 36). However, in our study, CARD8-AS1 had a higher expression level in normal astrocytes than in glioma cells, thus suggesting that it was a protective factor in glioma. The conflicting results on the role of CARD8-AS1 in tumors suggested that there may be a complex interactive functional network and need to be further studied. CRNDE has been reported to be an oncogenic lncRNA in cancers (38). Additionally, it has been observed to promote glioma growth and invasion *via* mTOR signalling and to promote malignant progression by attenuating the miR-384/PIWIL4/STAT3 axis (39, 40), in accordance with our results. LNCTAM34A was first named and described as an antisense RNA that could modulate the expression of the tumor suppressor microRNA-34a (miR34a) in multiple human tumors (41). In our study, we proved that LNCTAM34A is a tumor promotor in glioma. The glioma cells exhibited suppressed proliferation rates, reduced migration and lower EMT levels when LNCTAM34A was knocked down.

Moreover, our study found that 15 types of immune cell compositions between the two clusters were significantly different. TAMs had a larger population in Cluster 2 (with better survival) than in Cluster 1 (with worse survival). Monocytes, resting memory CD4 T cells and activated mast cells were more abundant in Cluster 1 than in Cluster 2. Moreover, we found that TAMs and monocytes had a significantly opposing correlation with risk scores. Specifically, TAMs were positively correlated with risk scores, and monocytes were negatively correlated with risk scores. Many studies have shown that macrophages can promote tumor progression and metastasis (42). As tumors progressed to malignancy, macrophages stimulated angiogenesis, enhanced tumor cell migration and invasion and suppressed antitumor immunity. In addition, M2, which is a subpopulation of macrophages, has been shown to play a vital role in the subversion of adaptive immunity and inflammatory circuits that promote tumor growth and progression (43). In glioma, macrophages were proven to facilitate tumor proliferation, survival and migration (44), and the high expression of M0 or M2 was related to a worsened overall survival time (45, 46). These previous findings supported our result that a high proportion of TAMs was associated with high-risk scores and poor prognoses in glioma patients. Monocytes are a type of nonadaptive immune cell that act as an essential regulator of cancer development and progression (47). Its different subtypes have opposing roles in promoting tumor growth and preventing cancer metastasis (48, 49). Furthermore, it can be recruited throughout the entire stage of tumor progression (50) and directly kill tumor cells *via* the cytokine‐mediated induction of cell death and phagocytosis (51). In addition, monocytes can also interact with adaptive immunity by directing the recruitment and function of lymphocytes within the tumor microenvironment (52). These previous studies explained the correlation between OS and monocytes (to a degree).

Although few studies have previously explored the relationship between m6A-related lncRNAs and the prognosis of some subtypes of glioma (18, 19), the choice of a specific part from glioma (instead of considering it as an entity to study) has apparent limitations. Due to the fact that glioma is a continuously progressing disease, the tumor grade of some gliomas gradually evolves from lower to higher grades (53–56). In addition, there were still limitations for the existing WHO glioma classification to accurately reflect all of the glioma patients’ malignant degrees. Our study focused on the relationship between m6A-related lncRNAs and the prognoses of glioma patients in all grades.

However, there were still some limitations in our study. For example, our glioma data were taken from the TCGA and CGGA databases. The model needs to be further validated in more glioma cohorts. In addition, only two of the nine m6A-related lncRNAs that were used to construct the prognostic signature had been tested for their expression levels in glioma cell lines *via* RT–PCR. The functions and interactions of other lncRNAs in glioma need to be further investigated.

## Methods

### Dataset Acquisition and m6A-Related lncRNA Screening

For the TCGA data, the RNA-Seq data of 698 gliomas, including glioblastoma (GBM) and lower grade glioma (LGG), as well as the corresponding clinicopathological data, were obtained from the TCGA website (https://portal.gdc.cancer.gov/). The TCGA dataset was randomly divided into the following two subsets: a training set (N=297) and a test set (N=283). This was done to construct and validate the prognostic signature in glioma, and the cases without clinical data matching were deleted. For the CGGA data, two subsets with RNA-Seq data and clinicopathological data, known as CGGA-seq-1 (N=280) and CGGA-seq-2 (N=657), were downloaded from the CGGA website (http://www.cgga.org.cn/). The RNA-Seq data of the normal tissue (N=423) were downloaded from the Genotype-Tissue Expression (GTEx) Project (http://gtexportal.org/home/) (**Table 2**).

**Table 2:** Information on multiple datasets.

Datasets	Samples		Type	Survival Information
	Glioma	Normal		
TCGA	698	0	Train set (n=297)	Partly*
	Test set (n = 283)
CGGA-seq-1	280	0	Test set	Yes
CGGA-seq-2	657	0	Test set	Yes
GTEx	0	423		No

*Survival data for 580 samples.

Based on previous studies, we extracted the expression profile of 23 m6A regulators from the TCGA database, including m6A writers (METTL3, METTL14, METTL16, WTAP, VIRMA, ZC3H13, RBM15 and RBM15B), readers (YTHDC1, YTHDC2, YTHDF1, YTHDF2, YTHDF3, HNRNPC, FMR1, LRPPRC, HNRNPA2B1, IGFBP1, IGFBP2, IGFBP3 and RBMX) and erasers (FTO and ALKBH5) (18, 20). Subsequently, a total of 14,086 lncRNAs were extracted from the TCGA dataset annotated by GENCODE (GRCh37). Moreover, we screened lncRNAs related to the 23 m6A regulators by using a Pearson correlation analysis and defined them as m6A-related lncRNAs. The correlation criteria were |cor value|>0.4 and p value<0.001. All of the analyses were performed and visualized by using R software.

### Clustering of Samples Based on the Expression Profile of m6A-Related lncRNAs

All of the gliomas from the TCGA database were clustered by using the Consensus Cluster Plus package according to the expression of m6A-related lncRNAs. The cumulative distribution function (CDF) of the consensus cluster was from k=2 to 9. The Kyoto Encyclopedia of Genes and Genomes (KEGG) pathway analysis was performed by using GSEA software. The immune cell infiltration characteristics were analysed by using the CIBERSORT package, and we defined the total of M0, M1 and M2 as tumor-associated macrophages (TAMs). The immune scores, stromal scores and estimate scores of the samples were calculated by using the estimate package in R software.

### Construction of the m6A-Related lncRNA Prognostic Signature

We screened the m6A-related prognostic lncRNAs by merging the survival data with the m6A-related lncRNA expression data and by performing correlation analyses in the training set. Next, we screened the differentially expressed (DE) m6A-related lncRNAs by comparing gliomas in the training set with normal tissues from the GTEx dataset. Significantly differential expression was defined as |log_2_FC|>1 and p<0.05. The m6A-related lncRNA with a high hazard ratio and positive fold change was defined as the tumor promotor. Conversely, the m6A-related lncRNA with a low hazard ratio and negative fold change was defined as a tumor suppressor. Seventy-three tumor promotors and 71 tumor suppressors were identified in glioma patients, and nine of them were used to construct the prognostic signature. These nine lncRNAs were selected *via* a LASSO regression analysis with a cut-off value of p<0.05. The following formula (based on a combination of the Cox coefficient and gene expression) was used to calculate the risk score.


$$Model:Risk\;score=\sum_{i=1}^{k}\beta iSi$$

βi represents the coefficients, and Si is the lncRNA expression level.

### Stratification and Validation of the Prognostic Signature

To evaluate the predictive ability of the MLPS for survival status, samples were classified into high-, mid- and low-risk groups by using the trisection points of all of the patients in the training set, and the corresponding risk scores were used to cut off the patients in the test set.

To test the correlation between the nine prognostic factor lncRNAs and the m6A regulators, we extracted the expression of these 9 lncRNAs and 23 m6A regulators in the TCGA and CGGA databases and detected their correlations by using the Pearson correlation analysis.

To stratify and validate the prognostic signature, samples were classified into high- or low-risk groups by using the medians of all of the risk scores. We further stratified patients into different subgroups according to age (age ≤ 60 or age>60), sex (male or female), WHO grade (lower-grade, including WHO I-II or higher-grade, including WHO III-IV), IDH status (mutant or wild type) and 1p/19q status (codeletion or noncodeletion). We classified patients aged ≤ 60 years as being younger and patients aged >60 years as being older in this study. The risk plot, survival curve and ROC curve were generated by using several packages in R software.

### Cell Culture and Transfection

The human glioma cell lines U87-MG, LN229 and U343 were purchased from ATCC (Manassas, VA). Human normal SVGP12 astrocytes were purchased from Shanghai Institutes for Biological Sciences (Shanghai, China). Cells were maintained in DMEM (Invitrogen, Carlsbad, CA) supplemented with 10% foetal calf serum (Gibco BRL) and 1% penicillin plus streptomycin (HyClone, Logan, UT) and incubated in a humidified incubator (37°C, 5% CO2). LNCTAM34A siRNAs were purchased from JTS Scientific and transfected by using Lipofectamine 2000 (Invitrogen, USA) according to the manufacturer’s instructions.

### Real-Time Polymerase Chain Reaction

Total RNA was extracted from cells by using TRIzol reagent (Invitrogen, Waltham, Massachusetts). Real-time polymerase chain reaction (RT–PCR) was performed by using the SYBR Green (Applied Biosystems, Foster City, CA) method with a CFX96 Real-Time PCR System (Bio–Rad, Hercules, California); GAPDH was used as the internal control. The settings for amplification were 95°C/120 s, followed by 39 cycles of 95°C/5 s and 60°C/30 s. GAPDH was used as an endogenous control, and the relative RNA expression was calculated by using the 2^−ΔΔCt^ method. Primers were generated by Sangon Biotechnology (Shanghai, China) (**Table 3**).

**Table 3:** Sequences of primers.

Gene	Primer	Sequence
CRDNE	Forwards	GCGGAGGTTAAGTGT
	Reverse	AACAGGTTTACCTCCTTATCTTCAGAA
LINC00641	Forwards	CAGCCTATACAGACAGCCC
	Reverse	CCAGTTGGTGCTGCCATTTG
LNCTAM34A	Forwards	AGCGGCATCTCCTCCACCTGAAA
	Reverse	TTGCCTCGTGAGTCCAAGGAGAAT
CARD-AS1	Forwards	TTCCTGACCTCAGCTGGAAT
	Reverse	GGGGAAAAACTCCACCCACAA
GAPDH	Forwards	GAGAAGGCTGGGGCTCATTT
	Reverse	AGTGATGGCATGGACTGTGG

### Cell-Counting Kit-8 (CCK8) Assay

For this assay, 5,000 U87 and LN229 cells were seeded in a 96-well plate. After the cells had been cultured for 0, 12, 36 or 48 hours, cell proliferation was measured *via* the Cell Counting Kit-8 (CCK-8; Dojindo, Tokyo, Japan) assay, according to the manufacturer’s instructions.

### Transwell Assay

The cell invasion ability was measured by using Transwell chambers (8-μm pore; BD Biosciences). U87 and LN229 cells (3× 10^4^ cells) suspended in 200 μl of serum-free culture medium were added to the upper chamber. The lower chamber was supplemented with DMEM supplemented with 10% FBS. After 24 h, the noninvading cells on the upper surface were separated, and the cells that had invaded to the bottom of the membrane were fixed with methanol and stained with 0.1% crystal violet. Digital image acquisition was performed after air drying. The number of invasive cells was counted by using a microscope.

### Western Blotting

U87-MG and LN229 cells were lysed by using RIPA lysis buffer (Thermo Scientific, Waltham, MA, USA). Total proteins were loaded into 10% SDS–PAGE gels and probed with E-cadherin (1:500, sc-7870, Santa), Vimentin (1:500, sc-32322, Santa) and β-Actin (1:5000, 3700s, CST), after which they were incubated with horseradish peroxidase-conjugated secondary anti-mouse IgG antibodies (1:2000; W4021, Beijing). The binding antibody was detected by using a hypersensitive ECL chemiluminescence kit (NCMBiotech), and images were collected by using a chemiluminescence imager (Image 800). Relative quantitative analysis was conducted based on the image band density ratio with ImageJ software (NIH, Bethesda, MD, USA).

### Statistical Analysis

A student’s t test, Pearson’s chi-square test and one-way ANOVA were used to compare different groups of data. Kaplan–Meier curves were used to evaluate the statistical significance of survival rates between different risk groups. The prediction accuracy of the risk characteristics was determined by using the ROC curve. Univariate and multivariate Cox regression analyses were performed to evaluate the significant prognostic factors. All of the statistical analyses were conducted by using R (ver. 5.0) and SPSS statistics (ver. 21) programs. P values<0.05 were considered to be statistically significant.

## Data Availability Statement

The original contributions presented in the study are included in the article/**Supplementary Material**. Further inquiries can be directed to the corresponding author.

## Author Contributions

HZ designed and corresponded for the work. YC and YG are co-first authors and contributed equally to this work. YC made the bioinformatic analysis in this study, performed biological experiment and was a major contributor in writing the manuscript. YG was a major contributor in draw figures, wrote part of the manuscript and revised the manuscript. XW, SL, and JX performed data analyses. WN, LM ,YQ, and MZ revised the manuscript and supervised the work. All authors contributed to the article and approved the submitted version.

## Funding

This research was funded by the National Key R&D Program of China (2019YFC1316104, FNL).

## Conflict of Interest

The authors declare that the research was conducted in the absence of any commercial or financial relationships that could be construed as a potential conflict of interest.

## Publisher’s Note

All claims expressed in this article are solely those of the authors and do not necessarily represent those of their affiliated organizations, or those of the publisher, the editors and the reviewers. Any product that may be evaluated in this article, or claim that may be made by its manufacturer, is not guaranteed or endorsed by the publisher.

## Supplementary Material

Supplementary Figure 1 Distribution of the 22 immune cells in the two clusters.

Supplementary Figure 2 The expression levels of CRNDE and LINC00641 in different stratifications. **(A)** The expression of CRNDE in the CGGA-seq-1 cohort and **(B)** in CGGA-seq-2 with different WHO grades, IDH mutation statuses, 1p/19q codeletion statuses and ages. **(C)** The expression of LINC00641 in the CGGA-seq-1 cohort and **(D)** in CGGA-seq-2 with different WHO grades, IDH mutation statuses, 1p/19q codeletion statuses and ages.

Supplementary Figure 3
**(A)** Kaplan–Meier survival curves of the CRNDE of primary glioma and recurrent glioma in the CGGA-seq-1 cohort and **(B)** in the CGGA-seq-2 cohort of the CGGA dataset, **(C)** Kaplan–Meier survival curves of the LINC00641 of primary glioma and recurrent glioma in the CGGA-seq-1 cohort and **(D)** in the CGGA-seq-2 cohort of the CGGA dataset. **(E)** The risk scores in different ethnic groups. **(F)** The survival curve of glioma patients stratified by age, sex, IDH status, WHO grade and 1p/19q status.

Supplementary Figure 4 The correlation between nine prognostic signature lncRNAs in MPLS and 23 m6A regulators in TCGA (below) and CGGA (upper) datasets.
