# Supplementary figures and images for: Identification of N6-Methyladenosine-Related lncRNAs as a Prognostic Signature in Glioma
Source: Front Oncol. 2022 Mar 3;12:789283. doi: 10.3389/fonc.2022.789283 (PMC8927984; doi:10.3389/fonc.2022.789283)

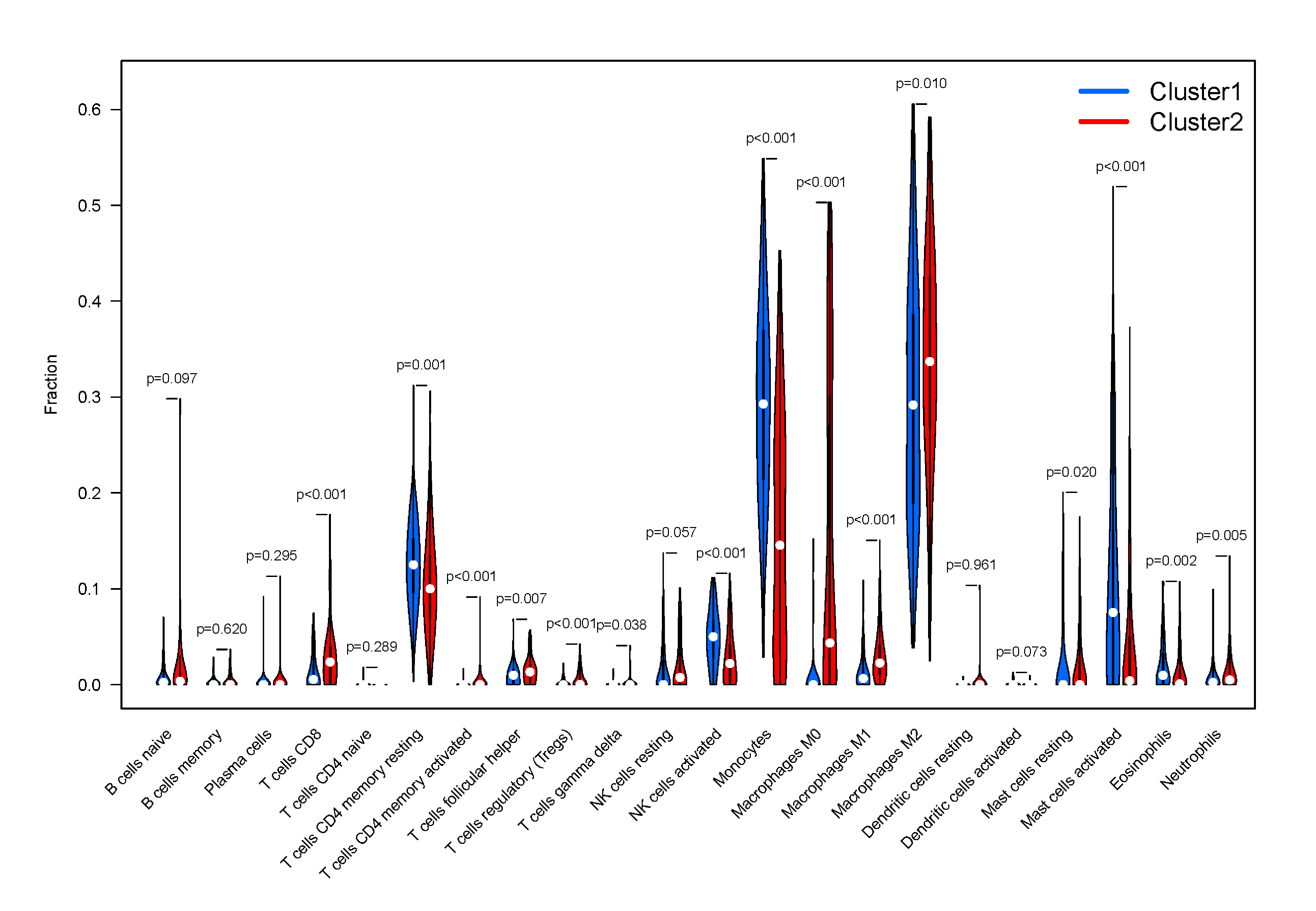

Supplement: Supplementary Figure 1 — Distribution of the 22 immune cells in the two clusters. [file Image_1.tif]

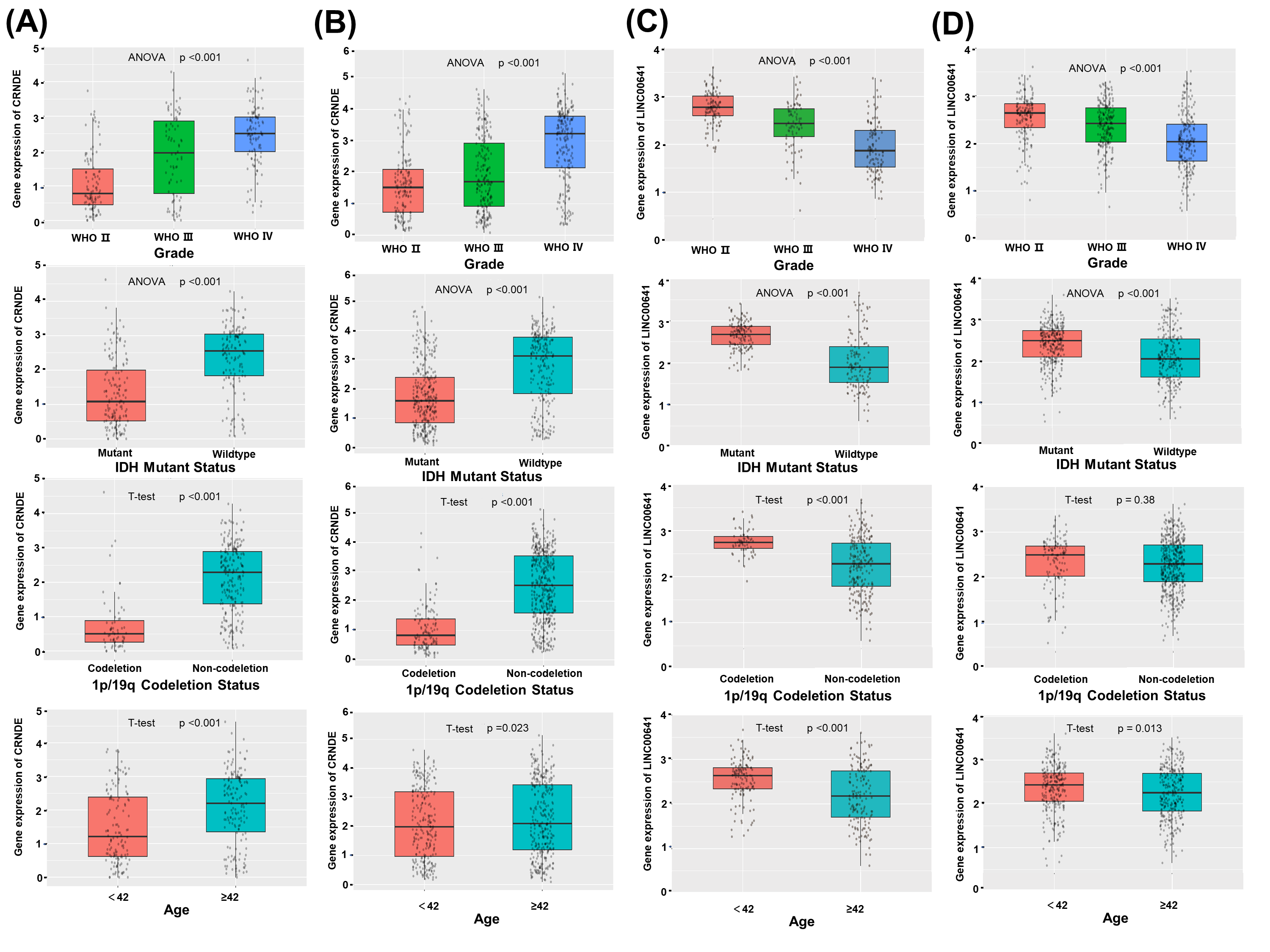

Supplement: Supplementary Figure 2 — The expression levels of CRNDE and LINC00641 in different stratifications. (A) The expression of CRNDE in the CGGA-seq-1 cohort and (B) in CGGA-seq-2 with different WHO grades, IDH mutation statuses, 1p/19q codeletion statuses and ages. (C) The expression of LINC00641 in the CGGA-seq-1 cohort and (D) in CGGA-seq-2 with different WHO grades, IDH mutation statuses, 1p/19q codeletion statuses and ages. [file Image_2.tif]

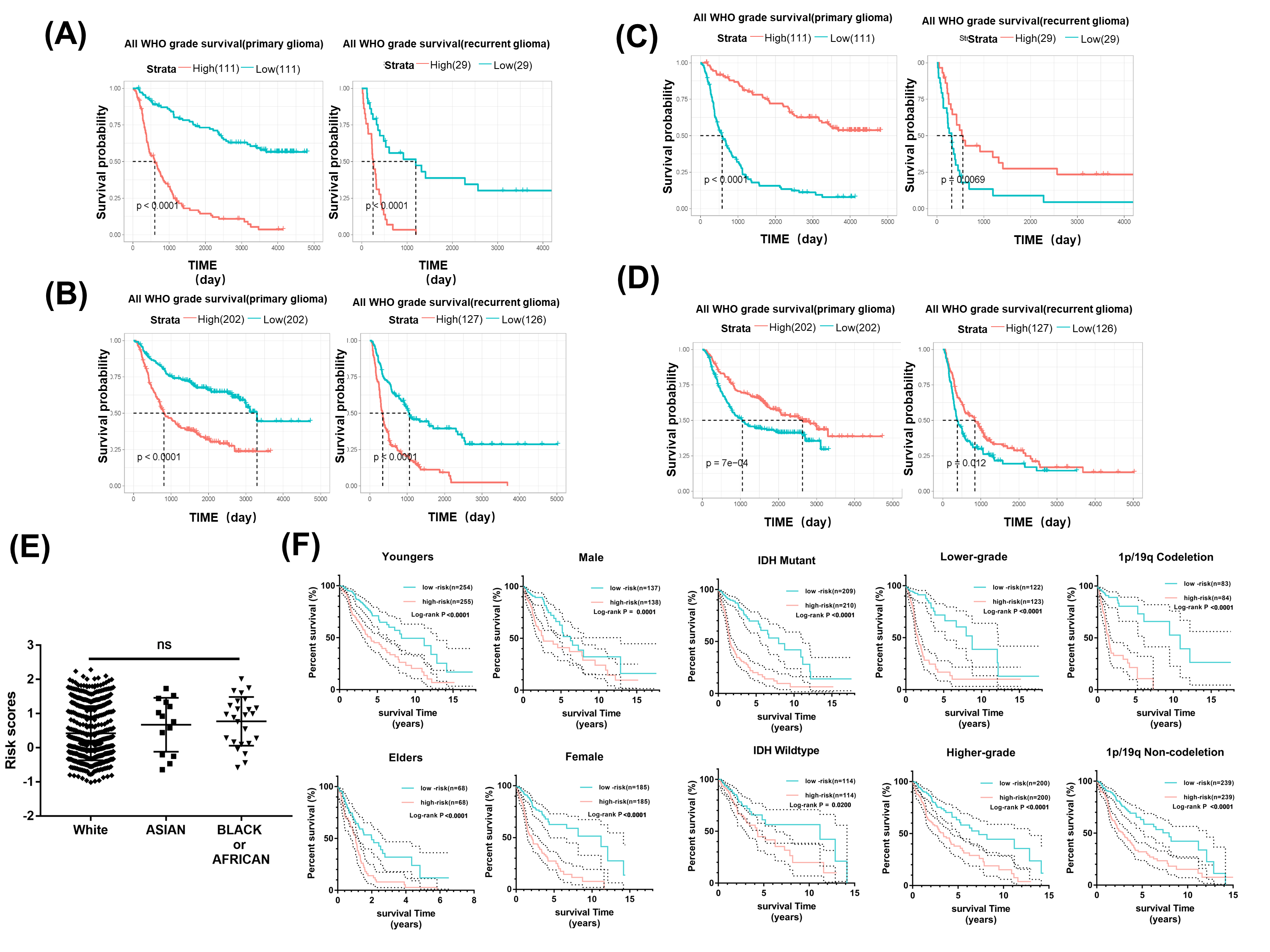

Supplement: Supplementary Figure 3 — (A) Kaplan–Meier survival curves of the CRNDE of primary glioma and recurrent glioma in the CGGA-seq-1 cohort and (B) in the CGGA-seq-2 cohort of the CGGA dataset, (C) Kaplan–Meier survival curves of the LINC00641 of primary glioma and recurrent glioma in the CGGA-seq-1 cohort and (D) in the CGGA-seq-2 cohort of the CGGA dataset. (E) The risk scores in different ethnic groups. (F) The survival curve of glioma patients stratified by age, sex, IDH status, WHO grade and 1p/19q status. [file Image_3.tif]

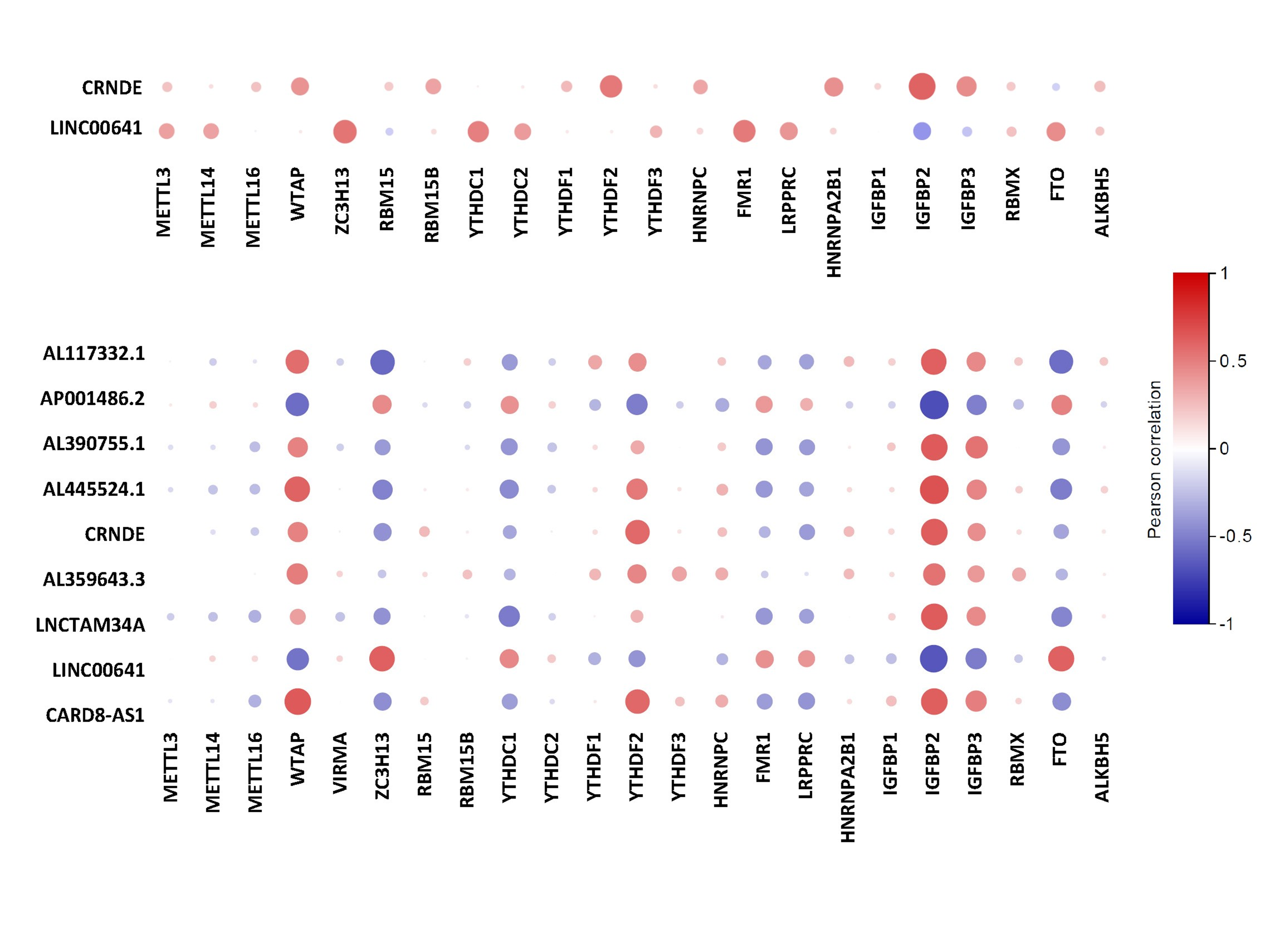

Supplement: Supplementary Figure 4 — The correlation between nine prognostic signature lncRNAs in MPLS and 23 m6A regulators in TCGA (below) and CGGA (upper) datasets. [file Image_4.tif]
