# Supplementary material for: Identification of N6-Methyladenosine-Related lncRNAs as a Prognostic Signature in Glioma
Source: Front Oncol. 2022 Mar 3;12:789283. doi: 10.3389/fonc.2022.789283 (PMC8927984; doi:10.3389/fonc.2022.789283)
Supplement: Supplementary file 5 [file Table_1.docx]

Table S1

The components of m6A RNA methylation regulators in writers. readers and erasers.

|  | Regulators |
| --- | --- |
| Writers | METTL3, METTL14, METTL16, WTAP, VIRMA, ZC3H13, RBM15, RBM15B |
| Readers | YTHDC1, YTHDC2, YTHDF1, YTHDF2, YTHDF3, HNRNPC, FMR1, LRPPRC, HNRNPA2B1, IGFBP1, IGFBP2, IGFBP3, RBMX |
| Erasers | FTO, ALKBH5 |
